# Neuroprotective, antimicrobial, antioxidant, chemotherapeutic, and antidiabetic properties of *Salvia Reuterana*: A mini review

**Published:** 2015

**Authors:** Elham Jafari, Sasan Andalib, Alireza Abed, Mahmoud Rafieian-Kopaei, Golnaz Vaseghi

**Affiliations:** 1 *Isfahann Pharmaceutical Sciences Research Center, School of Pharmacy and Pharmaceutical Science, Isfahan University of Medical Sciences, Isfahan, I. R. Iran*; 2 *Neurosciences Research Center, Imam Reza Hospital, Tabriz University of Medical Sciences, Tabriz, I. R. Iran *; 3 *Medicinal Plants Research Center, Shahrekord University of Medical Sciences, Shahrekord, I. R. Iran*; 4 *Applied Physiology Research Center, Isfahan University of Medical Sciences, Isfahan, I. R. Iran*

**Keywords:** *Salvia Reuterana*, *Neurological property*, *Antimicrobial property*, *Antioxidant property*, *Chemotherapeutic property*, *Antidiabetic property*

## Abstract

**Objectives:** Herbal medicine is known as a valid alternative treatment. *Salvia Reuterana*, which has been used in the Iranian traditional medicine, is mostly distributed in the central highlands of Iran. *Salvia Reuterana* is a medicinal herb with various therapeutic usages. The aim of the present review is to take account of pharmacological properties of *Salvia Reuterana*.

**Materials and Methods:** The present review summarizes the literature with respect to various pharmacological properties of *Salvia Reuterana*.

**Results:** *Salvia Reuterana* possesses neurological, antimicrobial, antioxidant, chemotherapeutic, and antidiabetic properties.

**Conclusions:** *Salvia Reuterana *can be used as an alternative for treatment of several disorders.

## Introduction

In the recent years we have witnessed rapidly increasing advances in the field of herbal medicine. World health organization (WHO) has reported that more than 80% of the world's population takes advantages of traditional medicine as their primary therapeutic needs on the grounds of their natural origin and low side effects. In the long history of the use of herbal medicines, human interactions with the environment have been thought to be a key element and it appears that systematic screening of medicinal herbs may discover novel effective compounds (Javidnia et al., 2009 ▶; Kamal & Jawaid, 2011 ▶).

*Salvia* is one of the largest genus of the Lamiaceae with over 900 species distributed throughout the world, especially in tropical and temperate areas (Masoud et al., 2011 ▶; Salimpour et al., 2011 ▶). 

In Iran, the genus *Salvia* (Sage) includes 58 species, of which 17 are known to be endemic. The name *Salvia *also refers to the Latin word Salvare, which means healer. Species of *Salvia *have traditionally been utilized in folk medicine to preclude stomach ailments and the common cold (Amiri, 2007 ▶; Naser Moadeli et al., 2013 ▶). Iranian traditional medicine have derived benefits of infusion and decoction of aerial parts of such genus as tonic, carminative, digestive, antispasmodic, and anti-inflammatory agents for treatment of diseases stemming from reactive oxygen species (ROS) (Karamian et al., 2013 ▶).

In addition to using Salvia species in cosmetics, perfumery, and the pharmaceutical industry, they have widely been utilized as herbal tea and food plant material flavoring (Nadaf et al., 2012 ▶). Such species contain monoterpene with antiseptic characteristics; even so, the compounds obtained from these species generate cellular DNA synthesis decline, a key factor in diagnosis and treatment of cancer (Masoud et al., 2011 ▶). The consequent metabolites, namely essential oils and plant extract of some Salvia species, are believed to exert antioxidant, antimicrobial, antifungal, anti-inflammatory, carminative, diuretic, hemostatic, spasmolytic, and aromatic effects (Sajjadi & Shahpiri, 2004 ▶). For example, aerial parts of *Salvia officinalis, Salvia sclare, Salvia macrosiphon,* *Salvia aegyptica, and Salvia Reuterana (S. Reuterana) *have been used as hypoglycemic, tonic, antimicrobial, anti-inflammatory, and anti-anxiety herbal drugs, respectively (Rabbani et al., 2005 ▶). Phytochemical assessments have revealed that some species of *Salvia* contain phenolic acids, phenolic glycosids, flavonoids, anthocyanins, coumarins, polysaccharides, strols, terpenoid, and essential oils (Amiri, 2007 ▶; Salimpour et al., 2011 ▶). It has been demonstrated that morphological characters of six *Salvia *species, that is, *Salvia atropatana, Salvia oligophylla, Salvia aethiopis, Salvia sclraea, S. Reuterana, *and* Salvia macrosiphon* are similar particularly in their leaves, trichomes, and flowers (Salimpour et al., 2011 ▶).

*S. Reuterana *Boiss refers to a perennial herb growing in the central highlands of Iran. The plant is known as Mariam Goli Esfahani in Persian. Evidence form a study on volatile constituents of* S. Reuterana *suggested that 21 components existed in the oil of the plant. It was shown that (E)-beta-ocimene (32.3%), alpha-gurjunene (14.1%), germacrened-D (11.2%), and hexyl acetate (7.6%) were the major constituents (Mirza & Sefidkon, 1999 ▶). Morphological and phytochemical diversity of intra- and inter- population of the plant have been assessed in some areas of Iran to domesticate this valuable plant (Masoud et al., 2011 ▶). Furthermore, pharmacological studies have recently attempted to evaluate anti-anxiety (Andalib et al., 2011 ▶; Gilhotra & Dhingra, 2008 ▶; Kalia et al., 2012 ▶; Rabbani et al., 2005 ▶; Rabbani et al., 2011 ▶; Verma et al., 2010 ▶), hypnotic (Andalib et al., 2011 ▶), and antibacterial (Javidnia et al., 2009 ▶) properties of the plant. The present review summarizes the literature reported by far regarding the neurological, antimicrobial, antioxidant, chemotherapeutic, and antidiabetic properties of *S. Reuterana*.

**Pharmacological properties of** ***S. Reuterana***

*Use of* *S. Reuterana extract in treatment of anxiety*

Anxiety, which is a generalized mood condition without an identifiable triggering stimulus, is known as a common disorder in todays' communities. It was shown that this disorder may last many years, and corresponds to significant personal distress, decreased quality of life, and increased morbidity and mortality (ref). Main drawbacks of anxiety drug therapy include co-morbid psychiatric disorders and increase in the medication dose, giving rise to deleterious side effects. Recently, there has been an increasing interest in assessing plants employed for treatment of sleep disorders and related diseases in traditional and alternative systems of medicine (Gilhotra & Dhingra, 2008 ▶; Kalia et al., 2012 ▶; Verma et al., 2010 ▶). Anxiolytic effect of *S. Reuterana *was shown by Rabbani et al. (Rabbani et al., 2005 ▶). The authors demonstrated that hydroalcoholic extract (100 mg/kg, po) exerted anxiolytic-like effect in the elevated plus maze, and anxiety level declined in EPM model test in mice. The extracts showed sedative impact that was much lower than diazepam in the locomotor activity test (Rabbani et al., 2005 ▶). The sedative property was further supported by its effects upon locomotor activity bringing about sedation at a dose of 100 mg/kg (Rabbani et al., 2005 ▶). It was also shown that doses lower than 100 mg/kg did neither alter the locomotor activity nor exerted a remarkable anxiolytic impact (Rabbani et al., 2005 ▶). Moreover, Vaseghi et al., reported that the sedative effect of *S. Reuterana *extract (100 mg/kg) was much lower than those exerted by diazepam, elsewhere (Vaseghi et al., 2013 ▶). This showed a better profile as an anxiolytic medicine. The authors also argued that increase in dose of the extract may increase sedative properties but not anxiolytic effects (Vaseghi et al., 2013 ▶). The anxiolytic mechanism was postulated to affect GABA receptors (Kamal & Jawaid, 2011 ▶; Rabbani et al., 2005 ▶; Rabbani et al., 2011 ▶).

*Hypnotic effect of* *S. Reuterana* *for treatment of insomnia*

Insomnia is a common disorder characterized by difficulty falling asleep giving rise to multiple nervous problems. It has been reported that 10 to 20 percent of adults suffer from chronic insomnia. Interest has recently been focused on utilizing medicinal herbs to abate insomnia owing to deleterious side effects of synthetic sleep medicines. In Iranian folk medicine, *Coriandrum sativum, Salvia leriifolia, S. Reuterana*, and* Stachys lavanduli folia *have been utilized against insomnia. The aerial parts of *S. Reuterana *have traditionally been used on the grounds of its sedative and anxiolytic properties. The mechanism of inhibition of acetylcholinesterase by constituents of *S. Reuterana, *which is responsible for its sedative effects, was supported by Perry et al. (Perry et al., 2003 ▶). It was also reported that Miltirone inhibits the binding of [3H] flunitrazepam to central benzodiazepine receptor.

*Use of S. Reuterana extract as antimicrobial agents*

Given the fact that bacterial infections are serious health problems, a considerable amount of literature has been published with regard to new antimicrobial drugs from various sources (e.g., microorganisms, animals, and plants). Broadly speaking, it is difficult to ignore plant extracts and essential oils as potentially useful sources of antimicrobial compounds. The crude herbal extracts of aromatic plants have traditionally been used for food preservation and also as antimicrobials (Batooli et al., 2013 ▶; Javidnia et al., 2009 ▶; Sonboli et al., 2006 ▶). Compared to current antimicrobials, herbal antimicrobial compounds inhibit bacterial growth through different mechanisms. Such property may be clinically valuable against resistant microbial strains (Javidnia et al., 2009 ▶).

Javidnia et al. assessed antimicrobial effect of methanolic extracts of various parts of eleven indigenous wild plant species used in Iranian folk medicines for treatment of infection of nine species of microorganisms, namely *Escherichia coli, Staphylococcus aureus, Staphylococcus epidermis, Pseudomonas aeruginosa, Klebsiella pneumoniae, Salmonella typhi, Bacillus subtilis, Aspergillus niger, *and *Candida albicans* (Javidnia et al., 2009 ▶). By means of disk diffusion (0.5, 1, 2, and 4 mg/disk) and minimal inhibition concentration (MIC), the antimicrobial efficacy was assessed and *Salvia* species showed the most active antimicrobial property (Javidnia et al., 2009 ▶)*.*

*Use of S. Reuterana extract as a chemotherapeutic agent*

Medicinal herbs are important sources of potential chemotherapeutic agents used to treat cancers. Over 400 compounds have been obtained from plants using cytotoxicity bioassay. For instance, vinblastin, vincristine, etoposide, and taxol are plant-derived compounds that are accredited as anti-cancer drugs. A majority of these chemotherapeutic drugs exert anti-cancer impact through arresting cells at different stages of the cell cycle and subsequently causing apoptotic cell death. Interestingly, most of these agents possess exclusively a limited anti-solid tumor activity and exert a host of side effects. Investigations on new anti-cancer drugs with a higher potency and specificity against cancer cells have great values in medicine (Amirghofran et al., 2010 ▶; Amiri, 2007 ▶; Javidnia et al., 2009 ▶). Antitumor activity of several plants embodying* Salvia *species against different cell lines namely, Hela (Cervix epitheloid carcinoma), Raji (Burkitt's lymphoma), Fen (bladder carcinoma), K562 (myelogenous leukemia), and Jurkat (T cell leukemia), which were obtained from Iranian cell bank, was investigated by Amirghofran et al. (Amirghofran et al., 2010 ▶). The authors argued that, compared to other herbs, *Salvia *possesses more cytotoxic activity against tumor cells, which is due to the presence of various cytotoxic compounds in different *Salvia *species. Salvicine, salvinal, and tanshinone are cases in point. This compound has been utilized for treatment of breast cancer. The highest inhibitory effect of *Salvia* species was observed in *S. Reuterana *extract. In spite of the fact that the inhibitory effect of the extract on Jurkat, K562, Fen, and Hela cells failed to reach 50%, a strong inhibitory effect was seen on the Raji cell line as 50% of Raji cells growth was inhibited by the extract (21±0.8 μg/ml) (Amirghofran et al., 2010 ▶). The authors also suggested *S. Reuterana* for further investigations to discover the effective anti-cancer component (Amirghofran et al., 2010 ▶).

*Use of S. Reuterana extract as an antioxidant agent*

Oxidative stress includes a situation wherein a serious imbalance exists between the generations of reactive oxygen species (ROS) and antioxidant defense mechanisms, and gives rise to potential tissue dysfunction and damage. ROS are produced in the early and advanced stages of glycation. The glycation phenomenon is attributed to the nonenzymatic and non-oxidative covalent linkage of glucose molecule to protein. It was demonstrated that non-enzymatic protein glycation initially occurs by the creation of a Schiff base between amino groups of the protein chain and the aldehyde (or keto) groups of the sugars. Afterwards, the Schiff base enters a number of reactions to produce fluorescent brown pigments that is called advanced glycation products (Esmaeili et al., 2010 ▶; B Nickavar, 2012; Bahman Nickavar et al., 2007). The antioxidant property of methanolic extract of *S. Reuterana* was assessed by Esmaeili et al. using various established *in vitro *systems involving ferric reducing antioxidant power (Esmaeili et al., 2010 ▶), Trolox equivalent antioxidant capacity assay, and scavenging of 1,1-diphenyl-2-picrylhydrazyl radical (Esmaeili et al., 2010 ▶; Ghomi et al., 2012 ▶; Bahman Nickavar et al., 2007). The authors confirmed the highest scavenging activity in methanolic extract of *S. Reuterana* (15.01±1.00 µg/ml) (Esmaeili et al., 2010 ▶). 

Moreover, inhibitory effect of the plant on glycation- and oxidation-dependent damages to albumin induced by fructose was studied (Esmaeili et al., 2010 ▶). The methanolic extract derived from the aerial parts of *S. Reuterana*, as a polyphenolicrich extract, exerted a high inhibitory effect upon formation of AGEs (advanced glycation end products) up to 53.3% with a concentration of 50 µg/ml as the same concentration of aminoguanidine (AG) that leaded to 60.4% inhibition, using different *in vitro* glycation models (Esmaeili et al., 2010 ▶). Methanolic extract of the plant also showed a significant decline in protein carbonyl formation and increase in protein thiol levels and protected structural changes of BSA (bovine serum albumin) from glycation processes, which may pertain to its antioxidative activity (Esmaeili et al., 2010 ▶).

*α-Amylase inhibitory activities of S. Reuterana*

α-Amylase refers to one of the enzymes contributing to catalyzing the breakdown of starch to maltose and subsequently to glucose, the only sugar able to be consumed by the body. The inhibition of such enzymes diminishes blood glucose level. Herbs and their constitutions have recently attracted much attention because they can abate diabetes, and a host of researchers has worked on hypoglycemic agents from medicinal plants. The evidence from the published literature suggests that polyphenols and flavonoids are among natural active antidiabetic compounds. It has been demonstrated that such compounds possess various biological properties, involving carbohydrate hydrolyzing enzyme inhibition and antioxidant activity. Nickavar et al. studied α-Amylase inhibitory activities of ethanol extracts derived from six Iranian *Salvia *species by means of in vitro model (Bahman Nickavar et al., 2010). It was shown that an increase in graded concentrations of the *S. Reuterana *extract diminished α-amylase activity from 1.19±0.45 to 25.01±1.68%. Nevertheless, the inhibitory activity of *S. Reuterana *on α-amylase was weak and failed to reach the 50% inhibition level of enzyme activity (Bahman Nickavar et al., 2010).

*Effect of S. Reuterana aerial parts on serum parameters in normal and streptozotocin-induced diabetic rats*

Eidi et al. assessed the effect of aerial parts of *Salvia Reuterana* upon serum parameters (i.e., glucose, triglycerides, total cholesterol, urea, uric acid, creatinine, aspartate aminotransferase (AST), and alanine aminotransferase (ALT)) in normal and diabetic rats (Eidi et al., 2012 ▶). Following oral treatment with S. Reuterana extract (0.25 and 0.5 g/kg body weight) and glibenclamide (standard antidiabetic drug) for a period of 14 days, a significant decline in serum glucose, triglycerides, total cholesterol, urea, uric acid, creatinine, AST, ALT, and increased plasma insulin in streptozotocin-induced diabetic rats were reported compared to normal rats. The authors concluded that extracts from this plant illustrate a dose-dependent activity that is comparable to the standard antidiabetic drug, glibenclamide (Eidi et al., 2012 ▶).

Medicinal plants are utilized by approximately 80% of the population of the world (ref). In spite of successes of synthetic drugs, they have a number of adverse effects. Natural remedies are becoming popular alternatives owing to fewer side effects (ref). Salvia is one of the largest genuses of the Lamiaceae including roughly 900 species throughout the world. *S. Reuterana *Boiss, which is known as Mariam Goli Esfahani in Persian, is a perennial herb growing in the central highlands of Iran (ref). Pharmacological studies have thus far confirmed its antibacterial, antifungal, carminative, antianxiety, and antioxidant properties. Neurological, antimicrobial, chemotherapeutic, antioxidant, α-amylase inhibitory**,** and antidiabetic properties of *S. Reuterana* extract have been demonstrated by recent studies (ref). The anxiolytic mechanism affects GABA receptors, while the mechanism of inhibition of acetylcholinesterase contributes to its sedative effects (ref). Methanolic extract of *S. Reuterana* also causes a significant decline in protein carbonyl formation and increase in protein thiol levels and protected structural changes of BSA (bovine serum albumin) from glycation processes and this may be involved in such antioxidative activity (ref). Little, however, is known about the active component and mechanism of the antimicrobial, chemotherapic**, **α-amylase inhibitory, and serum parameters’ lowering properties of *S. Reuterana*. Thus, further research regarding *S. Reuterana* would be of great value in discovering such unknowns.
